# The ramp and all-out exercise test to determine critical power: validity and robustness to manipulations in body position

**DOI:** 10.1007/s00421-021-04739-9

**Published:** 2021-06-18

**Authors:** Richie P. Goulding, Denise M. Roche, Simon Marwood

**Affiliations:** 1 Laboratory for Myology, Vrije Universiteit, O|2 Labgebouw, De Boelelaan 1108, 1081 HZ Amsterdam, The Netherlands; 2 School of Health Sciences, Liverpool Hope University, Liverpool, UK

**Keywords:** Critical power, All-out exercise, Power–duration relationship, Exercise testing, Performance

## Abstract

**Purpose:**

The purpose of the present study was to determine whether a contiguous ramp and all-out exercise test could accurately determine critical power (CP) in a single laboratory visit during both upright and supine cycle exercise.

**Methods:**

Healthy males completed maximal ramp-incremental exercise on a cycle ergometer in the upright (*n* = 15) and supine positions (*n* = 8), with task failure immediately followed by a 3-min all-out phase for determination of end-test power (EP). On separate days, participants undertook four constant-power tests in either the upright or supine positions with the limit of tolerance ranging from ~ 2 to 15 min for determination of CP.

**Results:**

During upright exercise, EP was highly correlated with (*R*^2^ = 0.93, *P* < 0.001) and not different from CP (CP = 221 ± 40 W vs. EP = 226 ± 46 W, *P* = 0.085, 95% limits of agreement − 30, 19 W). During supine exercise, EP was also highly correlated with (*R*^2^ = 0.94, *P* < 0.001) and not different from CP (CP = 140 ± 42 W vs. EP = 136 ± 40 W, *P* = 0.293, 95% limits of agreement − 16, 24 W).

**Conclusion:**

The present data suggest that EP derived from a contiguous ramp all-out exercise test is not different from the gold-standard method of CP determination during both upright and supine cycle exercise when assessed at the group level. However, the wide limits of agreement observed within the present study suggest that EP and CP should not be used interchangeably.

## Introduction

The relationship between power and time to task failure during cycle exercise over durations spanning ~ 2–30 min is well-described by a hyperbolic function (Poole et al. 2016). This power–time relationship is defined by two parameters: critical power (CP); representing the power asymptote of the hyperbola, and *W′*; the curvature constant of the hyperbola representing a finite amount of work that can be performed above CP (Fukuba et al. 2003). CP represents an important parameter of aerobic function (Poole et al. 1988); separating exercise intensities where a steady state is attainable for pulmonary oxygen uptake ($\dot{V}$O_2_) and muscle metabolites (i.e. “heavy” intensity domain) from intensities where a steady state is unattainable (i.e. “severe” intensity domain) (Poole et al. 1988; Jones et al. 2008). During sustained exercise above CP, therefore, pulmonary $\dot{V}$O_2_ is driven towards its maximal value ($\dot{V}$O_2_ max) (Poole et al. 1988), intramuscular phosphocreatine projects towards a nadir (Jones et al. 2008), and exercise tolerance is predictably limited (Poole et al. 2016).

CP and *W′* are both sensitive to exercise training (Gaesser and Wilson 1988; Poole et al. 1990; Jenkins and Quigley 1992; Vanhatalo et al. 2008a) and offer an accurate prediction of endurance performance within the task duration range of ~ 2–30 min, underscoring the importance of these parameters as determinants of endurance performance. Moreover, elite male runners typically sustain 96% of their critical speed (analogous to CP) over the course of a marathon (Jones and Vanhatalo 2017), and critical speed is predictive of marathon performance across a range of abilities (Smyth and Muniz-Pumares 2020). CP and *W′* therefore provide invaluable information to endurance performance athletes, coaches and practitioners regarding the physiological and mechanical performance capabilities of an athlete, as well as the efficacy of a given training intervention or ergogenic aid. However, the conventional approach for establishment of CP and *W′* requires undertaking 3–5 constant load prediction trials to the limit of tolerance, ideally on separate days, such that confident estimates of the parameters may be obtained (Muniz-Pumares et al. 2018). Precise determination of CP is therefore both time- and labour-intensive for researchers and practitioners alike.

The power–duration relationship predicts that when *W′* has been fully depleted (i.e. at task failure), the highest power output that can be sustained is CP (Coats et al. 2003; Chidnok et al. 2013). Hence, Vanhatalo et al. (2007) demonstrated that during the final 30 s of an all-out 3-min bout of cycle exercise, power output plateaued to a work rate that was not different from, and highly correlated with, CP (i.e. end-test power; EP). More recently, Murgatroyd et al. (2014) demonstrated that CP could be accurately and reliably determined from the EP attained during a single exercise test, incorporating a 3-min all-out bout of exercise performed immediately following task failure during a maximal ramp-incremental exercise test, whereas *W′* was underestimated by the work above EP (WEP). This approach represents a significant advance over 1- (Clark et al. 2013) or 2-day (Vanhatalo et al. 2007; Bergstrom et al. 2012) testing procedures, because additional parameters of aerobic function (i.e., the gas exchange threshold, GET; mean response time of $\dot{V}$O_2_ kinetics; $\dot{V}$O_2max_) and thus the boundaries between moderate, heavy, and severe exercise intensity domains can be determined in a single visit. However, the validity of this test has not been confirmed by more than one study (Murgatroyd et al. 2014), nor have the robustness of its underlying principles been tested using alternative exercise modes. For instance, if EP from the contiguous ramp all-out test can be demonstrated to provide a valid estimate of CP and *W′* in an alternative mode of exercise (e.g. supine exercise), this would provide further evidence of the robustness of the underlying principles of this approach for the determination of CP. Such an approach would considerably reduce the burden associated with determination of the power-time parameters for sports and exercise practitioners.

The aim of this study was therefore to determine whether the contiguous ramp and all-out exercise test provides a valid estimate of CP during both upright and supine cycle exercise. It was hypothesised that (1) the EP derived from the ramp all-out exercise test would not be different from and highly correlated with CP during both upright and supine exercise, and consistent with previous research, (2) WEP derived from the ramp all-out exercise test would be different from *W′*.

## Methods

The data presented in the present study comprise retrospective analysis of data from five previous reports (Goulding et al. 2017, 2018a, b, 2019a, b). A total of 26 participants completed the upright exercise experiments (Goulding et al. 2017, 2018a, 2019b) and a total of 16 participants completed the supine exercise experiments (Goulding et al. 2018b, 2019a). However, several participants completed more than one of the original experiments, therefore in these instances only the data from the first experiment that the participant took part in was used for further analysis. Thus, 19 [mean ± standard deviation (SD); age: 28 ± 8 years; height: 181 ± 7 cm; body mass: 78 ± 9 kg] and 12 [age: 24 ± 5 years; height: 179 ± 9 cm; body mass: 79 ± 8 kg] healthy, recreationally active males took part in the upright and supine portions of the study, respectively, with 2 participants completing both upright and supine arms of the study. All participants provided written informed consent and all experiments received ethical approval from the Liverpool Hope University Research Ethics Committee. Participants were asked to avoid alcohol and strenuous exercise 24 h prior to each visit, not to consume caffeine 3 h prior to each visit, and to arrive 3 h postprandial. Tests were separated by at least 24 h, with each test performed at the same time of day (± 2 h).

### Equipment and measurements

All upright exercise tests were performed on an electronically braked cycle ergometer (Lode Excalibur Sport, Groningen, The Netherlands). Saddle height/angle and handlebar height/angle were recorded at the first test and replicated during each subsequent test. All supine exercise tests were performed with an electronically braked ergometric unit (Lode Angio, Groningen, The Netherlands) whilst lying flat on an Echo Cardiac Stress Table (Lode, Groningen, The Netherlands). Handrails were available for participants to grip throughout the tests to minimize backwards movement when forces were applied to the pedals, and an adjustable shoulder pad was positioned above the participant’s shoulders also to prevent backward movements. The participant’s feet were strapped securely to the pedals. The position between the shoulder pad and the distance between the hip and the crank were recorded and replicated during each visit. Both ergometers utilised in the present study featured cadence-dependent and cadence-independent power output controls, which was adjusted according to trial type (outlined below). Throughout all tests, pulmonary gas exchange and ventilation were measured breath-by-breath, with participants using a metabolic cart (Blue Cherry; Geratherm Respiratory, GmbH, Bad Kissingen, Germany). Participants wore a silicone facemask (Hans Rudolph, Kansas City, MO, USA) of known dead space attached to a low-dead space flow sensor (Geratherm Respiratory, GmbH, Bad Kissingen, Germany). Gas analysers were calibrated before each test using gases of known concentrations, and the flow sensors were calibrated using a 3-L syringe (Hans Rudolph, Kansas City, MO, USA).

### Exercise protocols

All tests were preceded by 3 min baseline cycling at 30 (for the ramp all-out test) or 20 W (for the constant work-rate tests). Participants were instructed to cycle between 70 and 90 rev min^−1^ (which was recorded and replicated in all subsequent visits). Task failure during the ramp portion of the ramp all-out test and the constant work-rate tests was defined as the point at which the cadence dropped below 70 (Goulding et al. 2017, 2018a, b) or 50 (Goulding et al. 2019a, b) rev min^−1^. Importantly, end-point cadences were the same for each test that a given individual performed to ensure consistency across comparisons, as these have been demonstrated to affect CP and *W′* (Green et al. 1995; Barker et al. 2006). Participants were given strong verbal encouragement throughout all tests.

### Ramp all-out test

Following an initial 3-min 30 W baseline period, power output increased linearly at a rate of 30 W min^−1^ (upright experiments) or 25 W min^−1^ (supine experiments) until task failure. These ramp rates were selected to elicit task failure in 8–12 min (Buchfuhrer et al. 1983). $\dot{V}$ O_2_ peak was taken as the highest 30 s value attained during the test and the GET was determined visually using established criteria (Beaver et al. 1986; Goulding et al. 2017, 2018a, b) after the test to enable estimation of appropriate work rates for the CP prediction trials. Following task failure, the cycle ergometer was immediately switched to its cadence-dependent (linear) mode, where the programmed linear factor determines flywheel braking resistance from work rate/cadence^2^. Hence, work-rate varies as a function of cadence (i.e. work-rate = linear factor × cadence^2^). Participants then immediately undertook an all-out effort for 3 min, as this duration has demonstrated to reliably result in a plateau in power output during the final 30 s (Burnley et al. 2006; Vanhatalo et al. 2007; Murgatroyd et al. 2014). Strong verbal encouragement was provided throughout the duration of the test to ensure that participants maintained their cadence as high as possible throughout the test. To prevent pacing, participants were not informed of elapsed time and cadence was obscured from vision at the onset of the all-out phase. CP is sensitive to variations in pedal cadence (Barker et al. 2006), however, in the ramp all-out test, flywheel resistance must be fixed before the test without prior knowledge of CP. It has previously been demonstrated that in 60 healthy young participants, CP was approximated by 3 × body mass (van der Vaart et al. 2014). Therefore, CP was estimated according to this relationship with the linear factor set such that at a cadence of 80 rev min^−1^, the estimated CP would be attained. CP is reduced during supine exercise (Goulding et al. 2017, 2018b, 2019a); therefore, for all supine tests, the estimated CP was taken as 2.5 times body mass. Power output during the all-out phase of the ramp all-out test was averaged into 30 s epochs to allow determination of the duration required to provide a stable EP. The power-time integral above EP during both the all-out and ramp phases (i.e. work done above end-test power; WEP) was determined to provide an estimate of *W′*.

### Constant work-rate tests

In each of the following four visits on separate days, a severe-intensity constant work-rate exercise test was undertaken to task failure to allow the determination of the power–duration relationship, each at different work rates. These work rates were estimated to be in the range of 50%Δ (i.e. 50% of the difference between GET and $\dot{V}$O_2max_)—110% $\dot{V}$O_2_ max, such that exercise durations ranged from 2 to 15 min for each subject (Poole et al. 2016). If exercise duration for a particular test fell outside this range, the work-rate was modified and the test repeated on a subsequent day. Work-rates were presented in random order. Following a 3-min 20 W baseline period, a step increase in work-rate was abruptly applied to the desired severe-intensity, and participants exercised until task failure was reached.

### Data analyses

CP and *W′* were determined by inputting power output (*P*), time to task failure (*T*) and work done (*W*) measured during the constant work-rate trials into three models: the hyperbolic power-time (Eq. 2), linear work-time (Eq. 3), and linear 1/*T* models (4):

1 $$P = W^{\prime}/T + {\text{CP}},$$

2 $$W = {\text{CP}} \times T + W^{\prime},$$

3 $$P = W^{\prime} \times \left( {1/T} \right) + {\text{CP}}.$$

The standard errors of the estimates (SEE) associated with *CP* and *W′* were expressed as a coefficient of variation (CV) relative to the parameter estimate. Best individual fit parameter estimates were obtained for each participant by selecting the model producing the lowest summed CV for both parameters.

### Statistical analysis

All data were analysed using SPSS Statistical Software (IBM SPSS Inc., Chicago, IL). One-way repeated measures ANOVA was used to determine differences in the 30 s epochs of $\dot{V}$O_2_ and power during the all-out phase. Planned repeated contrasts were used to locate significant differences. Paired samples *t* tests were employed to compare CP with EP and *W′* with WEP. Agreement between these parameters was assessed using Pearson’s correlation coefficient and Bland–Altman mean bias ± 95% limits of agreement (LoA). The coefficient of variation (CV; mean of both scores/standard deviation of both scores × 100) was also calculated to provide further information on agreement between these variables. 5% error for estimation of CP and 10% error associated with estimation of *W′* was set as the a priori threshold for accurate determination of these parameters, in line with current recommendations (Jones et al. 2019). Data are presented as mean ± SD unless otherwise stated; statistical significance was accepted at *P* < 0.05.

## Results

### Upright exercise

**Fig. 1:**
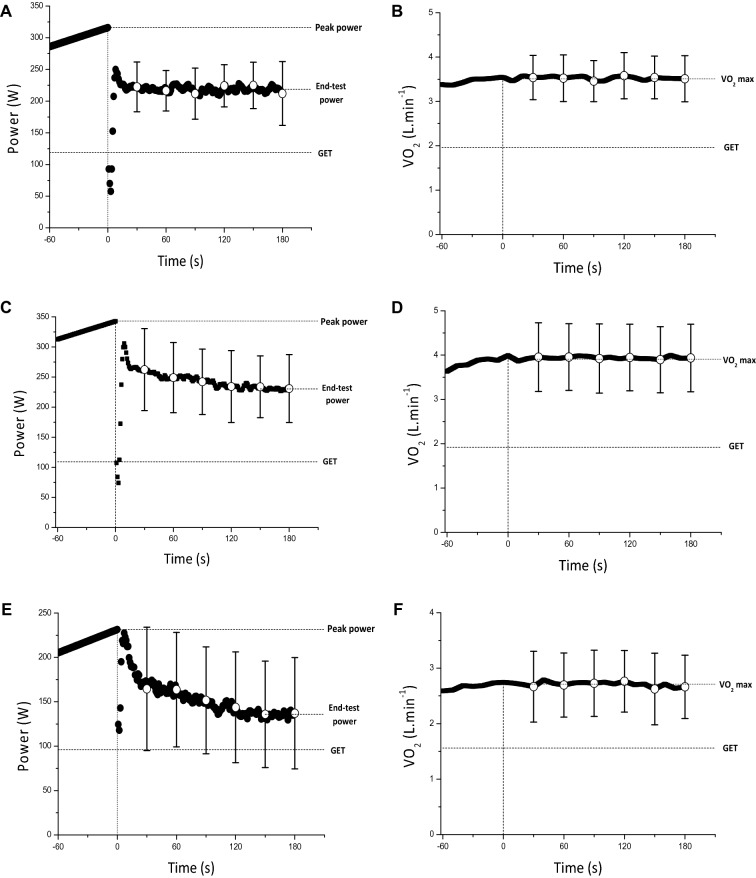
Group mean (black circles, with SD displayed as error bars) responses of power output (left) and $\dot{V}$O_2_ (right) during the ramp all-out test for negative pacing in the upright group (**A, B**), even pacing in the upright group (**C, D**) and the supine group (negative pacing only; **E, F**), plotted relative to the start of the 3-min sprint phase. Responses were averaged into 30 s epochs to facilitate comparisons. Following task failure during the ramp phase, the ergometer was switched from its cadence-independent mode to its cadence-dependent mode and participants performed a 3 min all-out exercise bout. * indicates significantly different from previous 30 s (*P* < 0.05)

**Fig. 2:**
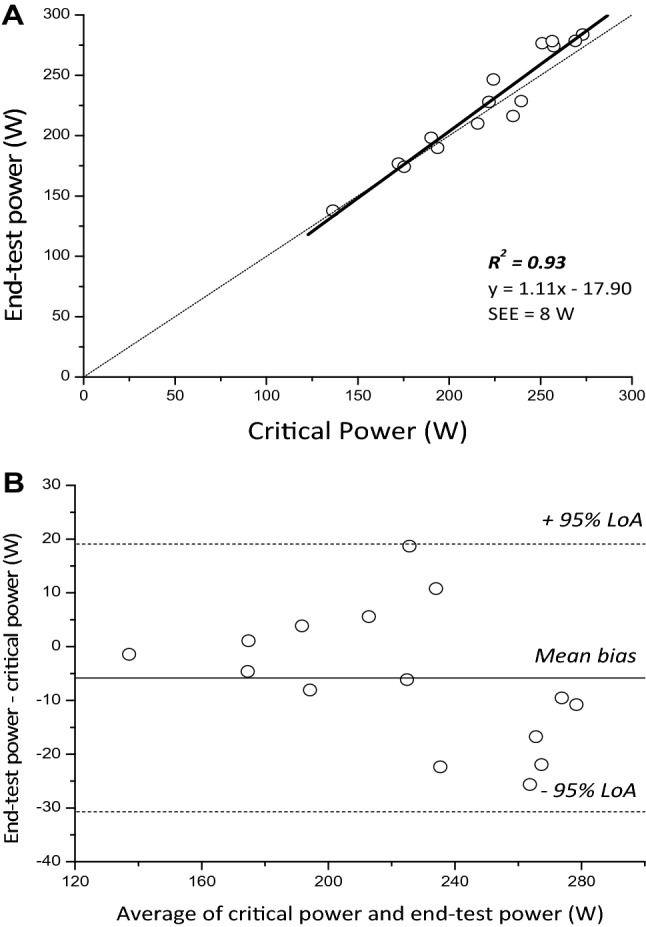
A linear regression (solid black line) demonstrating the degree of relative agreement between end-test power and critical power for the upright group (**A**). A Bland–Altman analysis showing the degree of agreement between the end-test power and critical power for each participant (**B**, clear circles), the mean bias (solid black line) and the 95% limits of agreement (LoA, dotted black lines) are also displayed

**Fig. 3:**
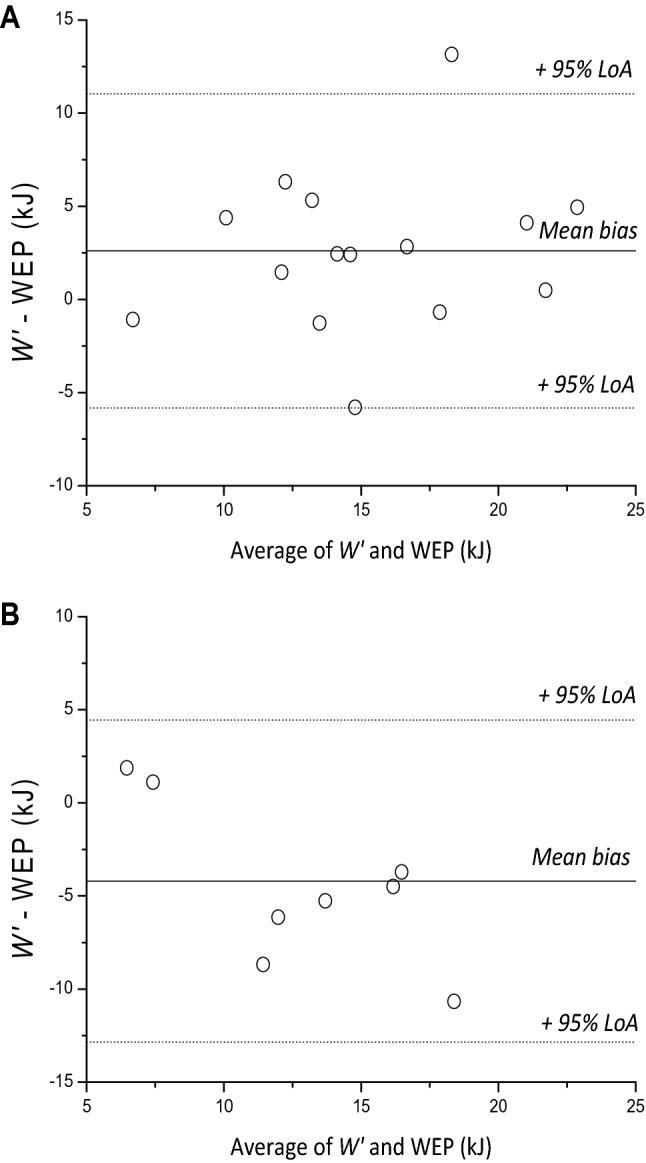
A Bland–Altman analysis showing the degree of agreement between *W′* and the WEP for each participant (clear circles) for the upright group (**A**) and the supine group (**B**). The mean bias is displayed as a solid black line, whereas dotted black lines indicate the 95% limits of agreement (LoA)

$\dot{V}$O_2_ during the all-out phase fell below 95% of $\dot{V}$O_2_ peak determined during the ramp phase in 4 participants, indicating a submaximal effort, therefore data are presented for the remaining 15 participants. In the remaining participants, $\dot{V}$O_2_ peak during the ramp phase (3.84 ± 0.69 L min^−1^) did not differ from the average $\dot{V}$ O_2_ measured across the all-out phase (mean: 3.80 ± 0.68 L min^−1^; *P* = 0.24) or the $\dot{V}$O_2_ peak from the constant work-rate trials (mean: 3.73 ± 0.71 L min^−1^; *P* = 0.339, Fig. 1). During the all-out phase, some participants demonstrated a decline in power with time (negative pacing strategy, *n* = 10, Fig. 1, *P* = 0.004), whereas the remaining participants demonstrated a lower power in the initial 30 s (*P* = 0.003) followed by a stable power from 30 to 180 s (even pacing strategy, *n* = 5, Fig. 1, *P* > 0.05). EP was therefore calculated as the average power over the final 30 s for negatively paced trials and the average power from 30 to 180 s for the evenly paced trials. EP and WEP averaged 226 ± 46 W and 14.0 ± 4.5 kJ, respectively; CP and *W′* averaged 221 ± 40 W (SEE: 7 ± 2 W) and 16.6 ± 5.4 kJ (SEE: 2.3 ± 1.2 kJ), respectively. CP was highly correlated with (*R*^2^ = 0.93, *P* < 0.001) (Fig. 2A), and not different from EP (*P* = 0.085, mean bias 6 W), with a CV between measures of 3 ± 2%. The LoA between the two parameters were − 30, 19 W (Fig. 2B), equivalent to -14, 9% of CP. WEP was lower than *W′* (*P* = 0.034, mean bias 2.6 kJ), with a typical error of 3.0 kJ and a CV between measures of 18 ± 14%. The 95% LoA between the two measures was -5.8, 11.0 kJ, equivalent to − 35.1, 66.4% of *W′* (Fig. 3).

### Supine exercise

**Fig. 4:**
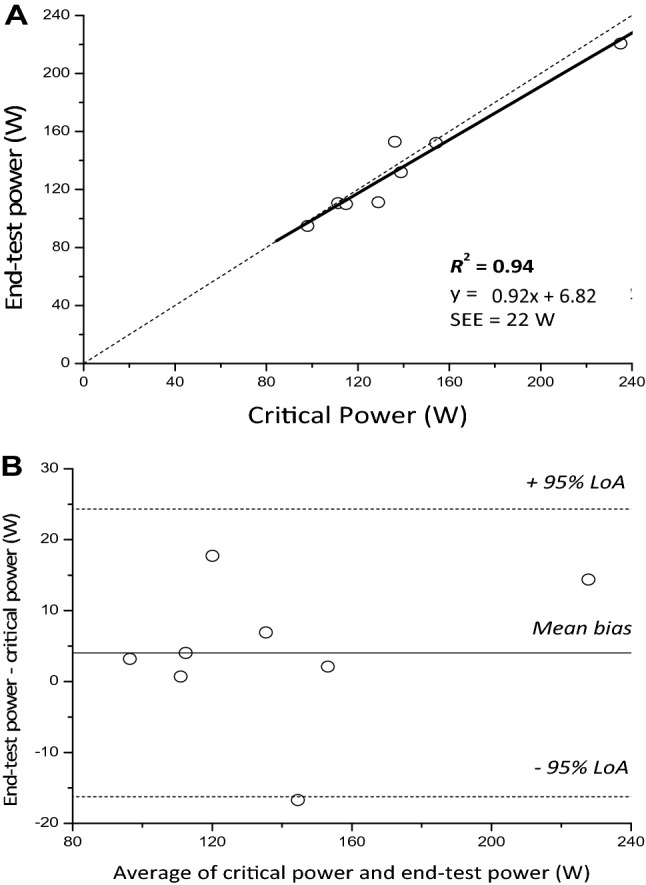
A linear regression (solid black line) demonstrating the degree of relative agreement between end-test power and critical power for the supine group (**A**). A Bland–Altman analysis showing the degree of agreement between the end-test power and critical power for each participant (**B**, clear circles), the mean bias (solid black line) and the 95% limits of agreement (LoA, dotted black lines) are also displayed

$\dot{V}$O_2_ during the all-out phase fell below 95% of $\dot{V}$O_2_ peak determined during the ramp phase in 4 participants, therefore data are presented hereafter for the remaining 8 participants. In the remaining participants, $\dot{V}$ O_2_ peak during the ramp phase (2.66 ± 0.48 L.min^−1^) did not differ from the average $\dot{V}$O_2_ measured across the all-out phase (mean: 2.71 ± 0.54 L min^−1^; *P* = 0.43) or the $\dot{V}$O_2_ peak from the constant work-rate trials (mean: 2.79 ± 0.58 L min^−1^; *P* = 0.18, Fig. 1). All participants in the supine group produced a negative pacing profile, therefore EP was taken as the average power over the final 30 s of the all-out phase (Fig. 1). EP and WEP averaged 136 ± 40 W and 14.8 ± 6.0 kJ, respectively; CP and *W′* averaged 140 ± 42 W (SEE: 4 ± 1 W) and 10.6 ± 3.3 kJ (SEE: 1.2 ± 0.7 kJ), respectively. CP was highly correlated with (*R*^2^ = 0.94, *P* < 0.001) and not different from EP (*P* = 0.293, mean bias 4 W), with a CV between measures of 4 ± 3% (Fig. 4A). The 95% LoA between the two measures was -16, 24 W (Fig. 4B), equivalent to − 11, 17% of CP. WEP was greater than *W′* (*P* = 0.031, mean bias − 4.6 kJ), with a CV between measures of 29 ± 14% (Fig. 3). The 95% LoA between the two measures was − 12.9, 4.4 kJ, equivalent to − 120.7, 41.8% of *W′*.

## Discussion

The present study determined whether a contiguous ramp all-out exercise test could determine CP and *W′* in a single laboratory visit during both upright and supine cycle exercise. The findings of the present study show that CP conventionally estimated from a series of severe-intensity prediction trials was not different from, and highly correlated with the EP from the contiguous ramp all-out exercise test during both modes of exercise, suggesting that the ramp all-out test may be used to determine CP in a single visit. However, WEP was significantly different from *W′* during both modes of exercise. This suggests that the ramp all-out exercise test is not appropriate for characterising *W′*, and thus, for instance, predicting endurance performance of 2–20 min duration. Furthermore, the wide 95% LoA (i.e. − 14, 9% of CP for upright, and − 11, 17% of CP for supine exercise) between CP and EP suggests that the two parameters should not be used interchangeably.

Murgatroyd et al. (2014) designed and validated the contiguous ramp all-out test utilised in the present study to enable the precise characterisation of the power–duration relationship in a single test. These authors demonstrated close agreement with CP determined via gold-standard procedures: EP and CP were not significantly different, and the 95% LoA between the two measures were ± 6% of CP. During exercise 10 W below EP in validation trials, all participants were able to complete 30 min of cycling and $\dot{V}$O_2_ attained a delayed steady state, whereas during exercise 10 W above EP, participants attained $\dot{V}$O_2_ max and task failure occurred within ~ 19 min (Murgatroyd et al. 2014). The purpose of the present study was therefore to independently validate these findings by determining whether EP could provide similarly precise estimates of CP during both upright and supine cycling. Our results are somewhat in agreement with those of Murgatroyd et al. (2014). Specifically, we found no difference between EP derived from the ramp all-out test and CP determined via gold-standard procedures in both body positions, and these parameters were highly correlated in both groups. Furthermore, the CV between EP and CP was less than the minimal level of error with which the gold-standard procedures used to determine CP are typically associated with in both body positions, i.e. 5% (Hill 1993; Mattioni Maturana et al. 2017). These findings therefore indicate a high level of agreement between EP and CP and suggest that the ramp all-out test is a valid tool to determine CP in a single laboratory visit. However, the 95% LoA between CP and EP were somewhat wide in both body positions (upright: − 30, 19 W or − 3 ± 11% of CP, supine: − 16, 24 W or 3 ± 15% of CP), a finding inconsistent with those of Murgatroyd et al. (2014). In this group of participants, therefore, it can be said that for any given individual there was a 95% probability that the difference between EP and CP would be between − 14% and + 9% (or − 12% and + 18% for supine exercise) of the mean of both measurements (Atkinson and Nevill 1998). This degree of error is somewhat larger than that typically deemed acceptable for estimation of CP with gold-standard modelling procedures (i.e. 5%, Hill 1993; Mattioni Maturana et al. 2017; Muniz-Pumares et al. 2018), therefore these finding suggest that CP and EP should not be used interchangeably. Vanhatalo et al. (2008a) previously demonstrated that a 4-week exercise training intervention resulted in a 25 W or 10% increase in CP. As the 95% LoA between EP and CP overlaps this change in both body positions in the present study, our data suggest that the ramp all-out test-determined EP is not sufficiently sensitive to monitor training-induced changes in CP. Moreover, 4/19 and 4/16 participants in the upright and supine positions were unable to maintain their $\dot{V}$O_2_ above 95% of their $\dot{V}$O_2_ peak during the all-out phase. This observation highlights the fact that, due to the difficulty of the ramp all-out test, participants must be highly motivated to complete it. However, given appropriate motivation, if the goal is to prescribe exercise within the heavy or severe domains, this may now be achieved in one test compared to the previously typical five (i.e. one incremental ramp test and four constant work-rate prediction trials), provided the tests are not in extremely close proximity to EP (i.e. not within ± 9–18% EP). The reason for the discrepancy regarding the 95% LoA between the present study and (Murgatroyd et al. 2014) is presently unclear, however, as no other study has examined the validity of the ramp all-out test.

WEP systematically underestimated *W′* during upright exercise, whereas WEP was greater than *W′* during supine exercise. The physiological determinants of *W′* are still not well understood (Poole et al. 2016), therefore the reasons for these discrepancies are not currently clear. However, Murgatroyd et al. (2014) also found that WEP during the all-out phase of the ramp all-out test was lower than *W′*, and the WEP during the standalone 3 min all-out test is typically lower than *W′* (Vanhatalo et al. 2007). Moreover, CP and *W′* derived from constant work-rate prediction trials have been shown to overestimate ramp exercise performance (Black et al. 2016), suggesting that *W′* may be reduced during ramp exercise. A reduction in *W′* in ramp and/or all-out exercise versus constant work-rate exercise could therefore potentially account for the underestimation of *W′* by WEP in the upright group in the present study. The reasons for the overestimation of *W′* by WEP in the supine position are less clear, but may simply be related to random error, given the 95% LoA between the two measurements was equivalent to 81.25% of *W′*. Irrespective of the reason for the overestimation of *W′* by WEP in the supine position, a major attraction of the power–time relationship is that once CP and *W′* are known, it becomes possible to predict endurance exercise performance with high precision for exercise durations spanning 2–30 min. However, the inability of WEP to provide accurate estimates of *W′* in the present study limits the use of this test to predict endurance performance.

## Limitations

One potential source of error that may have contributed to the wide 95% LoA in the present study is the fact that the linear factor must be set a priori before knowledge of a participant’s GET (Bergstrom et al. 2012; Clark et al. 2013). This is crucial because CP is sensitive to manipulations in cadence during variable power exercise (Vanhatalo et al. 2008b). The chosen linear factor must therefore be selected such that the cadence produced at the end of the all-out phase (i.e., when riding close to EP) elicits a power output that is close to the as yet unknown CP. In the present study, we utilized the same procedures as Murgatroyd et al. (2014), wherein the linear factor was set according to the previously determined relationship between CP and body mass (van der Vaart et al. 2014). In this study, CP approximated three times body mass in a cohort of 60 healthy active men; however, the relationship was inherently variable (*R*^2^ = 0.32). Variability in CP related to other factors, such as training status, is thus not considered in this estimation, and this likely translates to error in determination of CP from EP using the ramp all-out test. However, whilst these considerations likely explain a significant proportion of the error inherent in estimating CP from the ramp all-out test, our procedures were the same as those of Murgatroyd et al. (2014) and thus the reasons for the discrepancies between the data presented in that study and herein remain unclear. The protocol presented by (Constantini et al. 2014) would be a useful method to mitigate this concern, as these authors utilized a ramp and all-out exercise test separated by 20 min to determine CP and *W′*. This would allow time for estimation of the GET and thus selection of the appropriate linear factor for the all-out test. Future work in this area should validate this protocol to determine whether or not reliable and valid estimates of CP and *W′* can be obtained.

## Implications

CP and *W′* are highly relevant for athletic performance as they conflate to determine the tolerable duration of exercise over the range of 2–30 min. CP and *W′* can thus be used to prescribe training intensity, monitor the distribution of training intensity, predict competition performance, and monitor the performance of athletes over time. The ramp all-out test proposed by Murgatroyd et al. (2014), thus represents an attractive alternative to the previously typical 3–5 constant work-rate trials needed to precisely determine the power-time relationship. However, this is the first study to attempt to independently validate the ramp all-out test. The data presented herein suggest that the ramp all-out test may be used to prescribe heavy- or severe-intensity exercise for training or testing purposes, provided the intensities selected differ from EP by an appreciable margin (e.g. ± 9–18% EP using the data of the present study). For instance, it is common practice to prescribe heavy-intensity exercise as 40% of the difference between $\dot{V}$O_2_ max and the GET. However, this method does not take into account inter-individual variation CP and is thus liable to error. Using the ramp all-out test, greater confidence that a prescribed intensity is definitively heavy could be achieved by prescribing a work rate that is 50% of the difference between the GET and EP, for example. However, the wide LoA between CP and EP, along with the inability of WEP to predict *W′*, suggests that the test should not be used for performance prediction and/or athlete monitoring over time due to the unacceptably large error involved in estimation of the power–time relationship from the ramp all-out test. It is therefore recommended that traditional constant work-rate prediction trials are used in favour of the ramp all-out test in applied and practical settings for these purposes. Future research should test the robustness of these findings in various populations, e.g. athletes and patient populations.

## Conclusion

This study demonstrates that EP derived from a single, contiguous ramp all-out test was not different from and highly correlated with CP in both supine and upright exercise positions. Despite this, the 95% LoA between the two parameters was large, suggesting that EP derived from the ramp all-out test does not provide precise estimates of CP at the individual level. Moreover, WEP could not be used to estimate *W′*, limiting the practical utility of the ramp all-out test.

## Data Availability

Data are available upon request from the authors.

## Abbreviations

%Δ: Percentage difference between gas exchange threshold and maximal oxygen uptake
ANOVA: Analysis of variance
CP: Critical power
CV: Coefficient of variation
EP: End-test power
GET: Gas exchange threshold
LoA: Limits of agreement
*P*: Power
SD: Standard deviation
SEE: Standard error of the estimate
*T*: Time
$\dot{V}$O_2_: Pulmonary oxygen uptake
$\dot{V}$O_2max_: Maximal oxygen uptake
*W*: Work
*W*′: Work capacity available above critical power
WEP: Work performed above end-test power
